# An unexpected rhenium(IV)–rhenium(VII) salt: [Co(NH_3_)_6_]_3_[Re^VII^O_4_][Re^IV^F_6_]_4_·6H_2_O

**DOI:** 10.1107/S2056989019009757

**Published:** 2019-07-12

**Authors:** James Louis–Jean, Samundeeswari Mariappan Balasekaran, Adelheid Hagenbach, Frederic Poineau

**Affiliations:** a Department of Chemistry and Biochemistry, University of Nevada Las Vegas, 4505 South Maryland Parkway, Las Vegas, Nevada, 89154, USA; b Department of Chemistry and Biochemistry, Freie University Berlin, Berlin 14195, Germany

**Keywords:** crystal structure, hexa­mine-cobalt, perrhenate, hexa­fluoro­rhenate

## Abstract

The title hydrated salt, tris­[hexa­amminecobalt(III)] tetraoxidorhenate(VII) tetra­kis­[hexa­fluorido­rhenate(IV)] hexa­hydrate, arose unexpectedly due to possible contamination of the K_2_ReF_6_ starting material with KReO_4_. It consists of octa­hedral [Co(NH_3_)_6_]^3+^ cation (Co1 site symmetry 1), tetra­hedral [Re^VII^O_4_]^−^ anions (Re site symmetry 1) and octa­hedral [Re^IV^F_6_]^2−^ anions (Re site symmetries 1and ). The [ReF_6_]^2−^ octa­hedral anions (mean Re—F = 1.834 Å), [Co(NH_3_)_6_]^3+^ octa­hedral cations (mean Co—N = 1.962 Å), and the [ReO_4_]^−^ tetra­hedral anion (mean Re—O = 1.719 Å) are slightly distorted. A network of N—H⋯F hydrogen bonds consolidates the structure. The crystal studied was refined as a two-component twin.

## Abstract 2

The title hydrated salt, tris­[hexa­amminecobalt(III)] tetroxidorhenate(VII) tetra­kis­[hexa­fluorido­rhenate(IV)] hexa­hydrate, arose unexpectedly due to possible contamination of the K_2_ReF_6_ starting material with KReO_4_. It consists of octa­hedral [Co(NH_3_)_6_]^3+^ cation (Co1 site symmetry 1), tetra­hedral [Re^VII^O_4_]^−^ anions (Re1 site symmetry 1) and octa­hedral [Re^IV^F_6_]^2−^ anions (Re site symmetries, Re2: 1; Re3 and Re4: 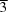). A network of N—H⋯F hydrogen bonds consolidates the structure.

## Chemical context   

The chemistry of Re^VII^ is dominated by the tetra­hedral perrhenate anion, [ReO_4_]^−^ (Latimer, 1952 ▸; Abram, 2003 ▸) while Re^IV^ is typically found in salts containing octa­hedral [Re*X*_6_]^2−^ (*X* = F, Cl, Br, I) anions (Berthold & Jakobson, 1964 ▸; Jorgensen & Schwochau, 1965 ▸; Grundy & Brown, 1970 ▸; Louis-Jean *et al.*, 2018 ▸). The salts of [Re*X*_6_]^2−^ (*X* = Cl, Br, I) can be prepared in high yield by the reduction of a perrhenate starting material in the corresponding concentrated H*X* acid (Briscoe *et al.*, 1931 ▸; Watt *et al.*, 1963 ▸). However, salts of [ReF_6_]^2−^ are typically prepared from the solid-state melting reaction of [Re*X*_6_]^2−^ (*X* = Cl, Br, I) with *A*HF_2_ (*A* = NH_4_^+^, K^+^) followed by an aqueous work-up (Ruff & Kwasnik, 1934 ▸; Louis-Jean *et al.*, 2018 ▸). Such a procedure is found to be challenging. Nonetheless, an improved procedure for the preparation of *A*_2_[ReF_6_] (*A* = K, Rb, Cs) salts as well as their X-ray single-crystal structures was recently reported (Louis-Jean *et al.*, 2018 ▸).
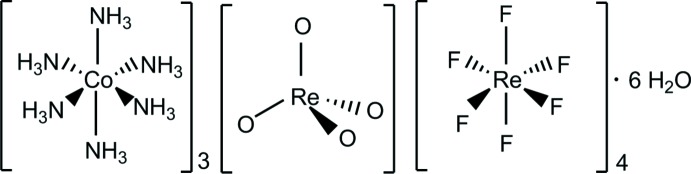


In the process of exploring the coordination chemistry of hexa­fluoro­rhenate(IV) compounds, the title compound (**I**), an unexpected mixed-valence rhenium(IV)–rhenium(VII) salt arose in an effort to prepare [Co(NH_3_)_6_]_2_[ReF_6_]_3_ by metathesis from K_2_[ReF_6_] and Co(NH_3_)_6_Cl_3_ in water (353 K). Yellow–orange needle-like crystals of (**I**) were obtained within two hours by slow evaporation in water at room temperature. The crystals of (**I**) are air stable over short periods, but decompose to a black material after six months of storage at ambient temperature.

## Structural commentary   

**Figure 1:**
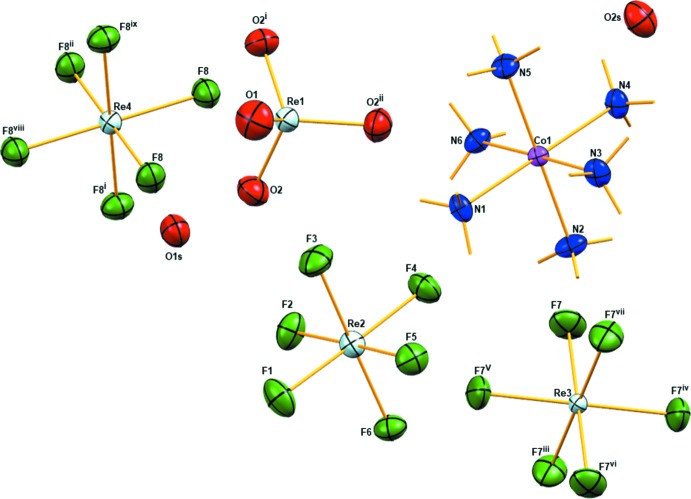
The mol­ecular structure of (**I**) showing displacement ellipsoids drawn at the 50% probability level for all non-H atoms. Symmetry codes: (i) 1 − *x* + *y*, 1 − *x*, *z*; (ii) 1 − *y*, *x* − *y*, *z*; (iii) 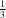 + *y*, 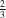 − *x* + *y*, 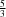 − *z*; (iv) 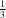 + *x* − *y*, −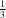 + *x*, 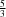 − *z*; (v) 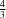 − *x*, 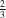 − *y*, 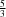 − *z*; (vi) 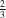 + *x* − *y*, 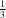 + *x*, 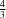 − *z*; (vii) −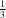 + *y*, 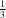 − *x* + *y*, 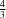 − *z*; (viii) 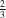 − *x*, 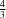 − *y*, 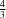 − *z*; (ix) −*x* + *y*, 1 − *x*, *z*; (*x*) 1 − *y*, 1 + *x* − *y*, *z*.

The structure of (**I**) (Fig. 1 ▸) is built up from a [Co(NH_3_)_6_]^3+^ cation, three distinct [ReF_6_]^2−^ anions, one [ReO_4_]^−^ anion, and two water mol­ecules of crystallization: these components are held together by electrostatic forces and hydrogen bonding. Site symmetries for the metal atoms are Co1: 1 (Wyckoff position 18*f*), Re1: 3 (Wyckoff position 6*c*), Re2: 1 (Wyckoff position 18*f*), Re3: 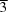 (Wyckoff position 3*a*), and Re4: 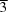 (Wyckoff position 3*b*).

The octa­hedral [Co(NH_3_)_6_]^3+^ cation in (**I**) is slightly distorted; the average Co—N bond length of 1.962 Å is in agreement with the average Co—N bond lengths of 1.963 Å in [Co(NH_3_)_6_](ReO_4_)·2H_2_O (Baidina *et al.*, 2012 ▸) and 1.966 Å in [Co(NH_3_)_6_](TcO_4_)_3_ (Poineau *et al.*, 2017 ▸). In (**I**), the shortest Co⋯Co and N⋯N separations between nearby [Co(NH_3_)_6_]^3+^ cations are 7.035 (1) and 4.473 (1) Å, respectively.

In the tetra­hedral [ReO_4_]^−^ anion in (**I)**, the average Re—O bond length (1.719 Å) is in agreement with the average Re—O bond length of 1.720 Å in [Co(NH_3_)_6_](ReO_4_)·2H_2_O (Baidina *et al.*, 2012 ▸). In (**I**) the values of three Re—O bond lengths, [Re1—O2^i^, Re—O2 and Re—O2^ii^ = 1.715 (8) Å; symmetry codes: (i) 1 − *x* + *y*, 1 − *x*, *z*; (ii) 1 − *y*, *x* − *y*, *z*] are slightly shorter than the fourth one [Re—O1 = 1.748 (14) Å]. In (**I**), all O—Re­—O bond angles in the [ReO_4_]^−^ anion are 109.5 (3)°. However, in [Co(NH_3_)_6_](ReO_4_)·2H_2_O, the [ReO_4_]^−^ anion is slightly distorted by up to 2.7° (Baidina *et al.*, 2012 ▸).

The [ReF_6_]^2−^ anions are slightly distorted, with Re—F bond lengths varying from 1.916 (6) Å to 1.929 (6) Å. All the Re—F bond lengths in the Re3- and Re4-centred anions are of equal distances of 1.952 (6) and 1.950 (6) Å, respectively, by symmetry. Overall, the average Re—F bond length (1.834 Å) in (**I)** is notably shorter than the average Re—F bond length (1.951 Å) in *A*_2_[ReF_6_] (*A* = K, Rb, Cs) salts previously studied (Louis-Jean *et al.*, 2018 ▸).

## Supra­molecular features   

**Figure 2:**
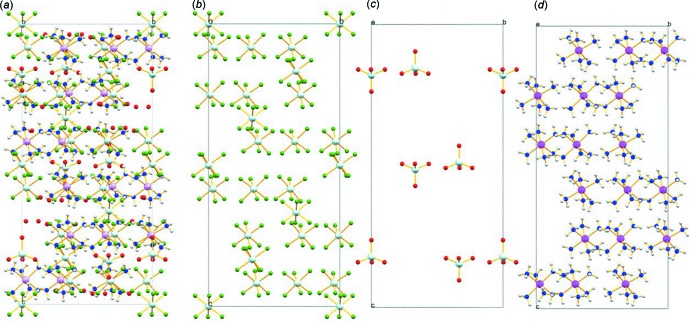
Unit-cell plots showing only (*a*) the complete structure of (**I**), (*b*) the [ReF_6_]^2−^ octa­hedra, (*c*) the [ReO_4_]^−^ tetra­hedra and (*d*) the [Co(NH_3_)_6_]^3+^ octa­hedra viewed along the crystallographic *b* axis. Color of atoms: Re aqua blue, F green, Co purple, N blue, O red, H gray.

**Figure 3:**
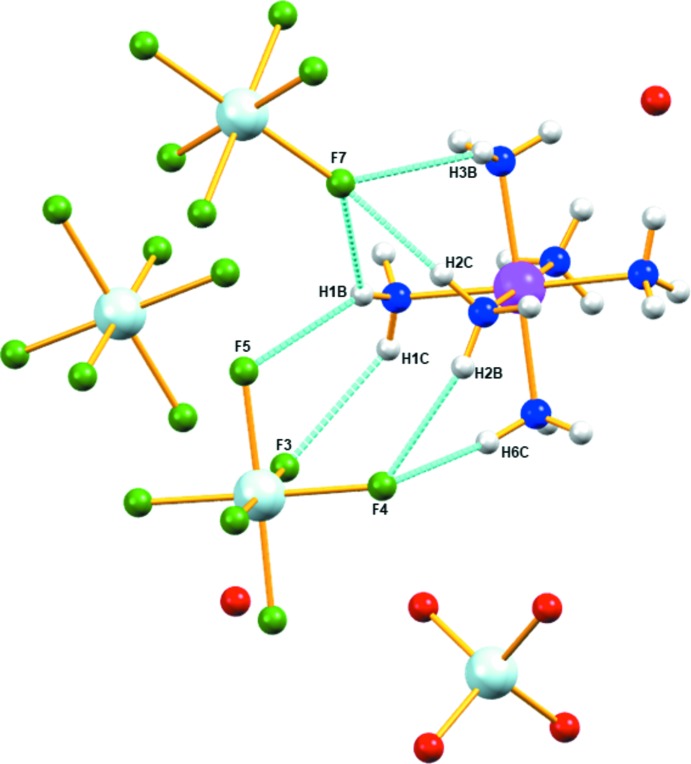
Detail of the hydrogen bonding (blue dotted lines) in (**I**). Atom colors as in Fig. 2 ▸.

A perspective view of the unit-cell plots for (**I**) and its component ions ([ReF_6_]^2−^, [ReO_4_]^−^, and [Co(NH_3_)_6_]^3+^) are shown in Fig. 2 ▸. In the supra­molecular structure of the title compound, the ammine ligands of the cations form numerous N—H⋯F and N—H⋯O hydrogen bonds with the fluorine atoms of [ReF_6_]^2−^ anions and the water mol­ecules (Table 1 ▸, Fig. 3 ▸).

## Database survey   

To the best of our knowledge, (**I**) is the only reported hexa­halogenorhenate–perrhenate structure containing both rhenium(IV) and rhenium(VII). It is noted that K_2_[ReF_6_] used for the preparation of (**I**) was not characterized before use and the presence of perrhenate in (**I)** may be due to the presence of K[ReO_4_] in the starting material. Efforts to isolate the technetium (Tc-99) derivative compound, [Co(NH_3_)_6_]_3_ [(Tc^(vii)^O_4_) (Tc^(iv)^F_6_)_4_] are in progress.

## Synthesis and crystallization   

All chemicals were obtained commercially from Sigma Aldrich® and used without any further purification. The starting material, K_2_[ReF_6_], was prepared following the method described in our previous publication (Louis-Jean *et al.*, 2018 ▸).

K_2_[ReF_6_] (114 mg, 0.3 mmol) was dissolved in 2 ml of hot water (353 K), and [Co(NH_3_)_6_]Cl_3_ (53.5 mg, 0.2 mmol) dissolved in 1 ml of  H_2_O was added. The solution was allowed to evaporate slowly at room temperature and yellow-orange needle-like crystals of (**I)** were obtained within two hours. The compound was washed with H_2_O (3 × 1 ml), followed by iso­propanol (3 × 1 ml) and then diethyl ether (3 × 1 ml). Single crystals of (**I)** were grown in H_2_O by slow evaporation at room temperature. Yield: *ca* 91%. The presence of perrhenate in (**I)** is probably due to the presence of K[ReO_4_] in the starting material (*i.e.* K_2_ReF_6_).

## Refinement   

Crystal data, data collection and structure refinement details are summarized in Table 2 ▸. The H atoms of the co-crystallized water mol­ecules could not be located in the present experiment.

## Supplementary Material

Crystal structure: contains datablock(s) global, I. DOI: 10.1107/S2056989019009757/hb7830sup1.cif

Structure factors: contains datablock(s) I. DOI: 10.1107/S2056989019009757/hb7830Isup2.hkl

CCDC reference: 1939234

Additional supporting information:  crystallographic information; 3D view; checkCIF report

## Figures and Tables

**Table 1:** Hydrogen-bond geometry (Å, °)

*D*—H⋯*A*	*D*—H	H⋯*A*	*D*⋯*A*	*D*—H⋯*A*
N1—H1*A*⋯F1^i^	0.89	2.34	3.085 (11)	141
N1—H1*A*⋯F5^i^	0.89	2.29	3.078 (10)	148
N1—H1*B*⋯F5	0.89	2.32	3.037 (10)	138
N1—H1*B*⋯F7	0.89	2.42	3.098 (11)	133
N1—H1*C*⋯F3	0.89	2.40	3.144 (10)	141
N1—H1*C*⋯O1*S* ^ii^	0.89	2.35	3.084 (12)	140
N2—H2*A*⋯F2^iii^	0.89	2.46	3.243 (10)	148
N2—H2*A*⋯O2^iii^	0.89	2.54	3.089 (12)	121
N2—H2*B*⋯F4	0.89	2.31	3.137 (12)	155
N2—H2*B*⋯F4^iii^	0.89	2.58	3.161 (10)	124
N2—H2*C*⋯F7	0.89	2.08	2.928 (10)	158
N3—H3*A*⋯F6^iv^	0.89	2.57	3.054 (10)	115
N3—H3*A*⋯O2*S*	0.89	2.26	3.027 (12)	145
N3—H3*B*⋯F5^iv^	0.89	2.52	3.132 (10)	127
N3—H3*B*⋯F7	0.89	2.32	2.911 (11)	123
N3—H3*C*⋯F5^i^	0.89	2.24	3.112 (10)	166
N4—H4*A*⋯F2^iii^	0.89	2.16	3.038 (10)	170
N4—H4*A*⋯F6^iii^	0.89	2.57	3.126 (10)	121
N4—H4*B*⋯F6^iv^	0.89	2.19	2.969 (10)	146
N4—H4*C*⋯F8^v^	0.89	2.21	3.019 (11)	150
N5—H5*A*⋯O2*S*	0.89	2.08	2.936 (12)	162
N5—H5*B*⋯F1^i^	0.89	2.20	3.057 (11)	162
N5—H5*C*⋯F1^ii^	0.89	2.49	3.019 (11)	119
N5—H5*C*⋯F2^ii^	0.89	2.43	3.261 (11)	157
N6—H6*A*⋯F4^iii^	0.89	2.55	3.110 (10)	122
N6—H6*A*⋯F6^iii^	0.89	2.16	3.037 (10)	168
N6—H6*B*⋯F2^ii^	0.89	2.16	2.968 (10)	151
N6—H6*B*⋯O1*S* ^ii^	0.89	2.67	3.227 (11)	122
N6—H6*C*⋯F4	0.89	2.14	2.982 (10)	159

Symmetry codes: (i) 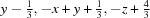; (ii) 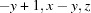; (iii) 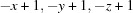; (iv) 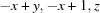; (v) 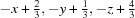.

**Table 2:** Experimental details

Crystal data	
Chemical formula	[Co(NH_3_)_6_]_3_[ReO_4_][ReF_6_]_4_·6H_2_O
*M*_r_	2030.40
Crystal system, space group	Trigonal, *R* 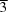
Temperature (K)	293
*a*, *c* (Å)	15.982 (3), 29.740 (5)
*V* (Å^3^)	6579 (2)
*Z*	6
Radiation type	Mo *K*α
μ (mm^−1^)	15.00
Crystal size (mm)	0.63 × 0.08 × 0.07

Data collection	
Diffractometer	Bruker D8 QUEST
Absorption correction	Numerical (Krause *et al.*, 2015 ▸)
*T*_min_, *T*_max_	0.02, 0.43
No. of measured, independent and observed [*I* > 2σ(*I*)] reflections	45169, 5223, 4885
*R*_int_	0.082
(sin θ/λ)_max_ (Å^−1^)	0.635

Refinement	
*R*[*F* ^2^ > 2σ(*F* ^2^)], *wR*(*F* ^2^), *S*	0.039, 0.114, 1.08
No. of reflections	5223
No. of parameters	189
H-atom treatment	H-atom parameters constrained
Δρ_max_, Δρ_min_ (e Å^−3^)	2.36, −4.16

Computer programs: *APEX3* and *SAINT* (Bruker, 2015 ▸), *SHELXT* (Sheldrick, 2015*a* ▸), *SHELXL2014* (Sheldrick, 2015*b* ▸) and *shelXle* (Hübschle *et al.*, 2011 ▸).

## supplementary crystallographic information

### Crystal data

**Table:**

[Co(NH_3_)_6_]_3_[ReO_4_][ReF_6_]_4_·6H_2_O	*D*_x_ = 3.075 Mg m^−^^3^
*M**_r_* = 2030.40	Mo *K*α radiation, λ = 0.71073 Å
Trigonal, *R*3	Cell parameters from 618 reflections
*a* = 15.982 (3) Å	θ = 3.2–32.0°
*c* = 29.740 (5) Å	µ = 15.00 mm^−^^1^
*V* = 6579 (2) Å^3^	*T* = 293 K
*Z* = 6	Rectangular box, translucent orange
*F*(000) = 5592	0.63 × 0.08 × 0.07 mm

### Data collection

**Table:**

Bruker D8 QUEST diffractometer	5223 independent reflections
Radiation source: sealed tube, Siemens KFFMo2K-90	4885 reflections with *I* > 2σ(*I*)
Curved graphite monochromator	*R*_int_ = 0.082
Detector resolution: 8.3333 pixels mm^-1^	θ_max_ = 26.8°, θ_min_ = 1.6°
'φ and ω scans'	*h* = −20→20
Absorption correction: numerical (Krause *et al.*, 2015)	*k* = −20→20
*T*_min_ = 0.02, *T*_max_ = 0.43	*l* = −37→37
45169 measured reflections	

### Refinement

**Table:**

Refinement on *F*^2^	0 restraints
Least-squares matrix: full	Hydrogen site location: inferred from neighbouring sites
*R*[*F*^2^ > 2σ(*F*^2^)] = 0.039	H-atom parameters constrained
*wR*(*F*^2^) = 0.114	*w* = 1/[σ^2^(*F*_o_^2^) + (0.0476*P*)^2^ + 166.9724*P*] where *P* = (*F*_o_^2^ + 2*F*_c_^2^)/3
*S* = 1.08	(Δ/σ)_max_ = 0.002
5223 reflections	Δρ_max_ = 2.36 e Å^−^^3^
189 parameters	Δρ_min_ = −4.16 e Å^−^^3^

### Special details

**Table:**

Geometry. All esds (except the esd in the dihedral angle between two l.s. planes) are estimated using the full covariance matrix. The cell esds are taken into account individually in the estimation of esds in distances, angles and torsion angles; correlations between esds in cell parameters are only used when they are defined by crystal symmetry. An approximate (isotropic) treatment of cell esds is used for estimating esds involving l.s. planes.
Refinement. Refined as a 2-component twin.

### Fractional atomic coordinates and isotropic or equivalent isotropic displacement parameters (Å^2^)

**Table:**

	*x*	*y*	*z*	*U*_iso_*/*U*_eq_	
Co1	0.30664 (8)	0.34382 (8)	0.58013 (3)	0.0133 (2)	
N1	0.4055 (5)	0.4205 (6)	0.6252 (2)	0.0215 (15)	
H1A	0.3824	0.3987	0.6526	0.032*	
H1B	0.4211	0.4822	0.623	0.032*	
H1C	0.4577	0.4154	0.6205	0.032*	
N2	0.3377 (7)	0.4637 (6)	0.5488 (3)	0.0257 (17)	
H2A	0.2965	0.4506	0.5262	0.038*	
H2B	0.3977	0.4909	0.538	0.038*	
H2C	0.3333	0.5041	0.5679	0.038*	
N3	0.2114 (6)	0.3556 (6)	0.6173 (3)	0.0244 (16)	
H3A	0.1533	0.3039	0.6133	0.037*	
H3B	0.2098	0.4085	0.6094	0.037*	
H3C	0.228	0.3597	0.6461	0.037*	
N4	0.2051 (6)	0.2661 (6)	0.5359 (3)	0.0236 (16)	
H4A	0.22	0.2963	0.5095	0.035*	
H4B	0.1488	0.2582	0.5454	0.035*	
H4C	0.2008	0.2086	0.5329	0.035*	
N5	0.2754 (6)	0.2239 (6)	0.6122 (3)	0.0255 (17)	
H5A	0.2133	0.1932	0.62	0.038*	
H5B	0.3118	0.2381	0.6367	0.038*	
H5C	0.2869	0.1861	0.5943	0.038*	
N6	0.4042 (6)	0.3341 (6)	0.5441 (2)	0.0220 (15)	
H6A	0.3851	0.3229	0.5155	0.033*	
H6B	0.4115	0.2859	0.5544	0.033*	
H6C	0.4602	0.3893	0.546	0.033*	
Re2	0.65656 (3)	0.62285 (3)	0.58421 (2)	0.02226 (14)	
F1	0.7514 (5)	0.7013 (5)	0.6281 (2)	0.0413 (16)	
F2	0.7555 (5)	0.6161 (5)	0.5496 (2)	0.0356 (14)	
F3	0.6309 (5)	0.5116 (5)	0.6198 (2)	0.0411 (16)	
F4	0.5603 (5)	0.5407 (5)	0.5415 (2)	0.0368 (14)	
F5	0.5608 (5)	0.6319 (5)	0.6196 (2)	0.0337 (14)	
F6	0.6801 (5)	0.7340 (4)	0.54917 (19)	0.0306 (12)	
Re1	0.6667	0.3333	0.51213 (2)	0.02289 (17)	
O1	0.6667	0.3333	0.5709 (5)	0.048 (4)	
O2	0.7663 (6)	0.4359 (6)	0.4929 (3)	0.0384 (17)	
Re4	0.6667	0.3333	0.8333	0.01831 (19)	
F8	0.5515 (4)	0.2843 (5)	0.7957 (2)	0.0336 (13)	
Re3	0.3333	0.6667	0.6667	0.01401 (18)	
F7	0.3284 (5)	0.5638 (5)	0.6295 (2)	0.0396 (15)	
O1S	0.7924 (6)	0.4886 (6)	0.6292 (3)	0.0379 (18)	
O2S	0.0654 (6)	0.1416 (6)	0.6190 (3)	0.0417 (19)	

### Atomic displacement parameters (Å^2^)

**Table:**

	*U*^11^	*U*^22^	*U*^33^	*U*^12^	*U*^13^	*U*^23^
Co1	0.0161 (5)	0.0146 (5)	0.0097 (5)	0.0079 (4)	0.0003 (4)	−0.0008 (4)
N1	0.019 (4)	0.028 (4)	0.014 (3)	0.009 (3)	−0.001 (3)	−0.003 (3)
N2	0.040 (5)	0.022 (4)	0.019 (4)	0.019 (4)	0.003 (4)	0.002 (3)
N3	0.027 (4)	0.030 (4)	0.018 (4)	0.015 (4)	−0.003 (3)	−0.009 (3)
N4	0.025 (4)	0.032 (4)	0.016 (4)	0.015 (3)	−0.005 (3)	−0.006 (3)
N5	0.029 (4)	0.024 (4)	0.022 (4)	0.013 (3)	0.002 (3)	0.006 (3)
N6	0.029 (4)	0.025 (4)	0.019 (4)	0.018 (3)	0.003 (3)	0.000 (3)
Re2	0.0227 (2)	0.0252 (2)	0.0173 (2)	0.01086 (15)	0.00121 (13)	0.00071 (13)
F1	0.033 (3)	0.051 (4)	0.026 (3)	0.010 (3)	−0.006 (3)	−0.006 (3)
F2	0.039 (3)	0.053 (4)	0.027 (3)	0.032 (3)	0.007 (3)	0.004 (3)
F3	0.044 (4)	0.038 (4)	0.045 (4)	0.023 (3)	0.009 (3)	0.018 (3)
F4	0.035 (3)	0.033 (3)	0.031 (3)	0.009 (3)	−0.007 (3)	−0.005 (3)
F5	0.035 (3)	0.038 (3)	0.030 (3)	0.019 (3)	0.010 (3)	0.004 (3)
F6	0.039 (3)	0.026 (3)	0.027 (3)	0.016 (3)	0.003 (3)	0.005 (2)
Re1	0.0219 (2)	0.0219 (2)	0.0248 (3)	0.01097 (11)	0	0
O1	0.058 (6)	0.058 (6)	0.028 (7)	0.029 (3)	0	0
O2	0.030 (4)	0.033 (4)	0.043 (4)	0.010 (3)	0.001 (3)	0.001 (3)
Re4	0.0199 (3)	0.0199 (3)	0.0151 (4)	0.00997 (13)	0	0
F8	0.030 (3)	0.033 (3)	0.034 (3)	0.013 (3)	−0.005 (3)	0.001 (3)
Re3	0.0135 (2)	0.0135 (2)	0.0151 (4)	0.00674 (12)	0	0
F7	0.042 (4)	0.034 (3)	0.043 (4)	0.019 (3)	0.003 (3)	−0.015 (3)
O1S	0.033 (4)	0.040 (4)	0.039 (4)	0.017 (4)	−0.006 (3)	−0.003 (3)
O2S	0.032 (4)	0.048 (5)	0.038 (4)	0.015 (4)	0.005 (3)	−0.004 (4)

### Geometric parameters (Å, º)

**Table:**

Co1—N2	1.958 (8)	N6—H6C	0.89
Co1—N6	1.960 (7)	Re2—F1	1.916 (6)
Co1—N1	1.965 (7)	Re2—F4	1.918 (6)
Co1—N3	1.965 (8)	Re2—F5	1.922 (6)
Co1—N5	1.968 (8)	Re2—F6	1.928 (6)
Co1—N4	1.972 (8)	Re2—F3	1.929 (6)
N1—H1A	0.89	Re2—F2	1.934 (6)
N1—H1B	0.89	Re1—O2^i^	1.715 (8)
N1—H1C	0.89	Re1—O2	1.715 (8)
N2—H2A	0.89	Re1—O2^ii^	1.715 (8)
N2—H2B	0.89	Re1—O1	1.748 (14)
N2—H2C	0.89	Re4—F8^iii^	1.952 (6)
N3—H3A	0.89	Re4—F8^iv^	1.952 (6)
N3—H3B	0.89	Re4—F8^ii^	1.952 (6)
N3—H3C	0.89	Re4—F8^v^	1.952 (6)
N4—H4A	0.89	Re4—F8	1.952 (6)
N4—H4B	0.89	Re4—F8^i^	1.952 (6)
N4—H4C	0.89	Re3—F7^vi^	1.950 (6)
N5—H5A	0.89	Re3—F7^vii^	1.950 (6)
N5—H5B	0.89	Re3—F7	1.950 (6)
N5—H5C	0.89	Re3—F7^viii^	1.950 (6)
N6—H6A	0.89	Re3—F7^ix^	1.950 (6)
N6—H6B	0.89	Re3—F7^x^	1.950 (6)
			
N2—Co1—N6	89.7 (4)	F1—Re2—F4	178.1 (3)
N2—Co1—N1	89.0 (4)	F1—Re2—F5	88.7 (3)
N6—Co1—N1	90.0 (3)	F4—Re2—F5	91.0 (3)
N2—Co1—N3	90.2 (4)	F1—Re2—F6	92.3 (3)
N6—Co1—N3	178.6 (3)	F4—Re2—F6	89.6 (3)
N1—Co1—N3	88.6 (3)	F5—Re2—F6	91.3 (3)
N2—Co1—N5	179.4 (3)	F1—Re2—F3	87.9 (3)
N6—Co1—N5	90.7 (4)	F4—Re2—F3	90.2 (3)
N1—Co1—N5	90.6 (4)	F5—Re2—F3	87.6 (3)
N3—Co1—N5	89.4 (4)	F6—Re2—F3	178.9 (3)
N2—Co1—N4	91.5 (4)	F1—Re2—F2	89.8 (3)
N6—Co1—N4	91.2 (3)	F4—Re2—F2	90.5 (3)
N1—Co1—N4	178.7 (3)	F5—Re2—F2	178.5 (3)
N3—Co1—N4	90.2 (3)	F6—Re2—F2	88.6 (3)
N5—Co1—N4	88.9 (4)	F3—Re2—F2	92.5 (3)
Co1—N1—H1A	109.5	O2^i^—Re1—O2	109.5 (3)
Co1—N1—H1B	109.5	O2^i^—Re1—O2^ii^	109.5 (3)
H1A—N1—H1B	109.5	O2—Re1—O2^ii^	109.5 (3)
Co1—N1—H1C	109.5	O2^i^—Re1—O1	109.5 (3)
H1A—N1—H1C	109.5	O2—Re1—O1	109.5 (3)
H1B—N1—H1C	109.5	O2^ii^—Re1—O1	109.5 (3)
Co1—N2—H2A	109.5	F8^iii^—Re4—F8^iv^	90.5 (3)
Co1—N2—H2B	109.5	F8^iii^—Re4—F8^ii^	180.0 (3)
H2A—N2—H2B	109.5	F8^iv^—Re4—F8^ii^	89.5 (3)
Co1—N2—H2C	109.5	F8^iii^—Re4—F8^v^	90.5 (3)
H2A—N2—H2C	109.5	F8^iv^—Re4—F8^v^	90.5 (3)
H2B—N2—H2C	109.5	F8^ii^—Re4—F8^v^	89.5 (3)
Co1—N3—H3A	109.5	F8^iii^—Re4—F8	89.5 (3)
Co1—N3—H3B	109.5	F8^iv^—Re4—F8	89.6 (3)
H3A—N3—H3B	109.5	F8^ii^—Re4—F8	90.5 (3)
Co1—N3—H3C	109.5	F8^v^—Re4—F8	180.0
H3A—N3—H3C	109.5	F8^iii^—Re4—F8^i^	89.5 (3)
H3B—N3—H3C	109.5	F8^iv^—Re4—F8^i^	180.0
Co1—N4—H4A	109.5	F8^ii^—Re4—F8^i^	90.5 (3)
Co1—N4—H4B	109.5	F8^v^—Re4—F8^i^	89.5 (3)
H4A—N4—H4B	109.5	F8—Re4—F8^i^	90.5 (3)
Co1—N4—H4C	109.5	F7^vi^—Re3—F7^vii^	91.0 (3)
H4A—N4—H4C	109.5	F7^vi^—Re3—F7	89.0 (3)
H4B—N4—H4C	109.5	F7^vii^—Re3—F7	89.0 (3)
Co1—N5—H5A	109.5	F7^vi^—Re3—F7^viii^	91.0 (3)
Co1—N5—H5B	109.5	F7^vii^—Re3—F7^viii^	91.0 (3)
H5A—N5—H5B	109.5	F7—Re3—F7^viii^	180.0 (4)
Co1—N5—H5C	109.5	F7^vi^—Re3—F7^ix^	180.0
H5A—N5—H5C	109.5	F7^vii^—Re3—F7^ix^	89.0 (3)
H5B—N5—H5C	109.5	F7—Re3—F7^ix^	91.0 (3)
Co1—N6—H6A	109.5	F7^viii^—Re3—F7^ix^	89.0 (3)
Co1—N6—H6B	109.5	F7^vi^—Re3—F7^x^	89.0 (3)
H6A—N6—H6B	109.5	F7^vii^—Re3—F7^x^	180.0
Co1—N6—H6C	109.5	F7—Re3—F7^x^	91.0 (3)
H6A—N6—H6C	109.5	F7^viii^—Re3—F7^x^	89.0 (3)
H6B—N6—H6C	109.5	F7^ix^—Re3—F7^x^	91.0 (3)

Symmetry codes: (i) −*x*+*y*+1, −*x*+1, *z*; (ii) −*y*+1, *x*−*y*, *z*; (iii) *y*+1/3, −*x*+*y*+2/3, −*z*+5/3; (iv) *x*−*y*+1/3, *x*−1/3, −*z*+5/3; (v) −*x*+4/3, −*y*+2/3, −*z*+5/3; (vi) *x*−*y*+2/3, *x*+1/3, −*z*+4/3; (vii) *y*−1/3, −*x*+*y*+1/3, −*z*+4/3; (viii) −*x*+2/3, −*y*+4/3, −*z*+4/3; (ix) −*x*+*y*, −*x*+1, *z*; (x) −*y*+1, *x*−*y*+1, *z*.

### Hydrogen-bond geometry (Å, º)

**Table:**

*D*—H···*A*	*D*—H	H···*A*	*D*···*A*	*D*—H···*A*
N1—H1*A*···F1^vii^	0.89	2.34	3.085 (11)	141
N1—H1*A*···F5^vii^	0.89	2.29	3.078 (10)	148
N1—H1*B*···F5	0.89	2.32	3.037 (10)	138
N1—H1*B*···F7	0.89	2.42	3.098 (11)	133
N1—H1*C*···F3	0.89	2.40	3.144 (10)	141
N1—H1*C*···O1*S*^ii^	0.89	2.35	3.084 (12)	140
N2—H2*A*···F2^xi^	0.89	2.46	3.243 (10)	148
N2—H2*A*···O2^xi^	0.89	2.54	3.089 (12)	121
N2—H2*B*···F4	0.89	2.31	3.137 (12)	155
N2—H2*B*···F4^xi^	0.89	2.58	3.161 (10)	124
N2—H2*C*···F7	0.89	2.08	2.928 (10)	158
N3—H3*A*···F6^ix^	0.89	2.57	3.054 (10)	115
N3—H3*A*···O2*S*	0.89	2.26	3.027 (12)	145
N3—H3*B*···F5^ix^	0.89	2.52	3.132 (10)	127
N3—H3*B*···F7	0.89	2.32	2.911 (11)	123
N3—H3*C*···F5^vii^	0.89	2.24	3.112 (10)	166
N4—H4*A*···F2^xi^	0.89	2.16	3.038 (10)	170
N4—H4*A*···F6^xi^	0.89	2.57	3.126 (10)	121
N4—H4*B*···F6^ix^	0.89	2.19	2.969 (10)	146
N4—H4*C*···F8^xii^	0.89	2.21	3.019 (11)	150
N5—H5*A*···O2*S*	0.89	2.08	2.936 (12)	162
N5—H5*B*···F1^vii^	0.89	2.20	3.057 (11)	162
N5—H5*C*···F1^ii^	0.89	2.49	3.019 (11)	119
N5—H5*C*···F2^ii^	0.89	2.43	3.261 (11)	157
N6—H6*A*···F4^xi^	0.89	2.55	3.110 (10)	122
N6—H6*A*···F6^xi^	0.89	2.16	3.037 (10)	168
N6—H6*B*···F2^ii^	0.89	2.16	2.968 (10)	151
N6—H6*B*···O1*S*^ii^	0.89	2.67	3.227 (11)	122
N6—H6*C*···F4	0.89	2.14	2.982 (10)	159

Symmetry codes: (ii) −*y*+1, *x*−*y*, *z*; (vii) *y*−1/3, −*x*+*y*+1/3, −*z*+4/3; (ix) −*x*+*y*, −*x*+1, *z*; (xi) −*x*+1, −*y*+1, −*z*+1; (xii) −*x*+2/3, −*y*+1/3, −*z*+4/3.
